# Comparative Study of Intravaginal Metronidazole and Triple-Sulfa Therapy for Bacterial Vaginosis

**DOI:** 10.1155/S1064744996000154

**Published:** 1996

**Authors:** Jack D. Sobel, Walter Chaim, Jessica Thomason, Charles Livengood, Richard Sweet, James A. McGregor, David Eschenbach, Sharon Hillier, Rudolph Galask, Sebastian Faro, Elizabeth Shangle, David Baker

**Affiliations:** 1 Department of Medicine and Obstetrics and Gynecology Wayne State University School of Medicine Detroit MI USA; 2 Department of Obstetrics and Gynecology Sinai Samaritan Medical Center Milwaukee WI USA; 3 Department of Obstetrics and Gynecology Duke University Medical Center Durham NC USA; 4 Department of Obstetrics and Gynecology San Francisco General Hospital San Francisco CA USA; 5 Department of Obstretrics and Gynecology University of Colorado Denver CO USA; 6 Department of Obstetrics and Gynecology University of Washington Seattle WA USA; 7 Department of Obstetrics, Gynecology and Reproductive Sciences University of Pittsburgh Pittsburgh PA USA; 8 Department of Obstetrics and Gynecology University of Iowa Hospitals and Clinics Iowa City IA USA; 9 Department of Gynecology and Obstetrics University of Kansas School of Medicine Kansas City KS USA; 10 Department of Obstetrics and Gynecology Loyola University Medical Center Maywood IL USA; 11 Department of Obstetrics and Gynecology SUNY Health Science Center Stony Brook NY USA; 12 Harper Professional Building Suite 2140 4160 John R Street Detroit MI 48201 USA

## Abstract

*Objective:* We sought to compare the efficacy of metronidazole gel vs. triple-sulfa cream in the treatment of bacterial vaginosis (BV).

*Methods:* In a double-blinded study, 247 women with symptomatic BV were randomly assigned to receive either 5 g of 0.75% metronidazole gel twice daily for 5 days or triple-sulfa cream twice daily for 5 days. There were 205 (96 treated with metronidazole and 109 treated with triple-sulfa) evaluable patients to compare efficacy at the final visit. Approximately 60% of these patients had been previously treated for BV, reflecting the recurrent nature of the disease in this patient population.

*Results:* At the first (12–16 days) return visit, 81/103 (79%) patients in the metronidazole group were cured compared with 80/113 (71%) patients in the triple-sulfa cream group (*P* = 0.333). At the final (28–35 days) return visit, 63/96 (66%) in the 96 metronidazole group remained cured compared with only 51/109 (47%) in the triple-sulfa group (*P* = 0.02). An intent-to-treat analysis similarly showed that the cure rate with metronidazole was superior to triple-sulfa (*P* ≤ 0.02). The clinical diagnosis demonstrated a high correlation (88%) with the diagnosis made by an independent assessment by Gram's stain. The side effects reported by the patients using metronidazole gel were infrequent and mild and were similar to those reported with triple-sulfa.

*Conclusions:* Metronidazole gel is a safe, effective, and well-tolerated treatment for BV.


Infectious Diseases in Obstetrics and Gynecology 4:66–70 (1996)
© 1996 Wiley-Liss, Inc.

### Comparative Study of Intravaginal Metronidazole and Triple-Sulfa Therapy for Bacterial Vaginosis

**Jack D. Sobel, Walter Chaim, Jessica Thomason, Charles Livengood, Richard Sweet, James A. McGregor, David Eschenbach, Sharon Hillier, Rudolph Galask, Sebastian Faro, Elizabeth Shangle, and David Baker**

*Departments of Medicine and Obstetrics and Gynecology, Wayne State University School of Medicine, Detroit, MI (J.D.S., W.C.); Department of Obstetrics and Gynecology, Sinai Samaritan Medical Center, Milwaukee, WI (J.T.); Department of Obstetrics and Gynecology, Duke University Medical Center, Durham, NC (C.L.); Department of Obstetrics and Gynecology, San Francisco General Hospital, San Francisco, CA (R.S.); Department of Obstretrics and Gynecology, University of Colorado, Denver, CO (J.A.M.); Department of Obstetrics and Gynecology, University of Washington, Seattle, WA (D.E.); Department of Obstetrics, Gynecology and Reproductive Sciences, University of Pittsburgh, Pittsburgh, PA (S.H.); Department of Obstetrics and Gynecology, University of Iowa Hospitals and Clinics, Iowa City, IA (R.G.); Department of Gynecology and Obstetrics, University of Kansas School of Medicine, Kansas City, KS (S.F.); Department of Obstetrics and Gynecology, Loyola University Medical Center, Maywood, IL (E.S.); Department of Obstetrics and Gynecology, SUNY Health Science Center, Stony Brook, NY (D.B.)*

### Abstract

*Objective:* We sought to compare the efficacy of metronidazole gel vs. triple-sulfa cream in the treatment of bacterial vaginosis (BV).

*Methods:* In a double-blinded study, 247 women with symptomatic BV were randomly assigned to receive either 5 g of 0.75% metronidazole gel twice daily for 5 days or triple-sulfa cream twice daily for 5 days. There were 205 (96 treated with metronidazole and 109 treated with triple-sulfa) evaluable patients to compare efficacy at the final visit. Approximately 60% of these patients had been previously treated for BV, reflecting the recurrent nature of the disease in this patient population.

*Results:* At the first (12–16 days) return visit, 81/103 (79%) patients in the metronidazole group were cured compared with 80/113 (71%) patients in the triple-sulfa cream group ($P = 0.333$). At the final (28–35 days) return visit, 63/96 (66%) in the 96 metronidazole group remained cured compared with only 51/109 (47%) in the triple-sulfa group ($P = 0.02$). An intent-to-treat analysis similarly showed that the cure rate with metronidazole was superior to triple-sulfa ($P \leq 0.02$). The clinical diagnosis demonstrated a high correlation (88%) with the diagnosis made by an independent assessment by Gram's stain. The side effects reported by the patients using metronidazole gel were infrequent and mild and were similar to those reported with triple-sulfa.

*Conclusions:* Metronidazole gel is a safe, effective, and well-tolerated treatment for BV.
© 1996 Wiley-Liss, Inc.

Key words

Vaginitis, vaginal infection, topical therapy

Since the first report established the efficacy of oral metronidazole for bacterial vaginosis (BV) in 1978,[1] oral metronidazole therapy has remained the most common treatment regimen. Numerous studies confirm the efficacy of metronidazole as divided-dose and single-dose therapy.[2–7] The cure

Address correspondence/reprint requests to Dr. Jack D. Sobel, Harper Professional Building, Suite 2140, 4160 John R Street, Detroit, MI 48201.

Received December 11, 1995

Accepted May 29, 1996

rates are high at both short-term (7–14 days) (75–90%) and long-term (28–30 days) (60–80%) follow-up.[4,8,9] The relatively few limitations in the use of oral metronidazole include concern about its use in the first trimester of pregnancy and allergy and gastrointestinal side effects.[10,11] Recently, alternative treatments for BV have become available with 2% clindamycin cream and 0.75% metronidazole gel. Several studies document the efficacy of intravaginal clindamycin.[12–15] However, efficacy reports with intravaginal 0.75% metronidazole have been few,[8,10,16] with only 1 placebo-controlled comparative study having been published to date.[9] In the present report, we describe a prospective, double-blinded comparative study of the efficacy of 0.75% metronidazole gel compared with intravaginal triple-sulfa (Sultrin, Ortho Pharmaceutical, Raritan, NJ).

### MATERIALS AND METHODS

### Patient Population

The patient population was composed of nonpregnant women, 18 years or older, attending the gynecologic clinics of 10 university health care or community clinics in the United States. The women were from diverse socioeconomic backgrounds. Approximately one-half of the patients were referred because of recurrent BV. A woman was eligible for enrollment in the study after a diagnosis of BV was determined, based on a modification of the 4 clinical criteria of Amsel et al.[17] These included the presence of ≥20% clue cells and at least 2 of the following 3 signs of vaginal discharge: a homogeneous appearance, a pH of ≥4.7, and a positive KOH test for volatile amines. The exclusion criteria included use of intrauterine contraceptive device, breast-feeding, ongoing anticoagulant therapy, presence of *Neisseria gonorrhoeae* or *Chlamydia trachomatis* cervicitis, coexistent *Candida* or *Trichomonas vaginalis* vaginitis, or cervical or vaginal signs of herpes simples virus infection. Further requirements for participation in the study were abstention from sexual intercourse during the treatment phase, use of non-lubricated condoms during the follow-up period, and abstention from vaginal douching and the use of any other intravaginal drug or cream. A written informed consent approved by the individual center's institutional review board was obtained from each patient before enrollment.

### Planned Sample Size

A sample size of 75 patients for each treatment group was determined for the analysis to have at least 80% power to detect a 30% difference in cure rates at 30 days following treatment. The actual sample size calculated was 38 patients for each treatment group for a 2-sided test with a $P$ of 0.05 and power of 80%.

### Blinding Method

The study was a parallel-group, double-blinded trial. The patients were assigned to therapy by randomization according to the patient numbers in individual centers.

### Treatment Method

The patients were given 5 g of metronidazole gel or triple-sulfa cream to be used twice daily for 5 days. The total dose of metronidazole for the treatment regimen was approximately 375 mg. Triple-sulfa cream is approved for the treatment of BV by intravaginal application twice daily for 4–6 days.

### Follow-Up and Evaluation

The treatment success or failure was determined at 2 follow-up visits. Success was defined by the absence of symptoms, a lack of 3 of the 4 clinical criteria defined by Amsel et al.,[17] and clue cells of <20% of the desquamated epithelial cells on the wet mount. A Gram's stain of the vaginal discharge was obtained at all visits to provide support of the diagnosis of BV and to serve as a quality-control assay. The Gram's stains were evaluated by an independent microbiologist blinded to the clinical diagnosis and treatment using parameters defined by Nugent et al.[18] The evaluations for efficacy and safety were made at visit 2 scheduled for 12–16 days and visit 3 scheduled for 28–35 days after completion of therapy.

### Statistical Analysis

The Cochran-Mantel-Haenszel statistical procedure and Fisher's exact test were used for comparisons in the data analysis.

### RESULTS

A total of 247 subjects were enrolled by 10 investigators, of these, 124 patients were assigned to receive 0.75% metronidazole gel and 123 were assigned to receive triple-sulfa cream. Seventy-eight (63%) of

124 patients in the metronidazole treatment group completed the study compared with 79/123 (64%) in the triple-sulfa treatment group. Twenty-five (20%) of 124 patients in the metronidazole group and 32/123 (26%) patients in the triple-sulfa group discontinued the study at visit 2 because of unsatisfactory therapeutic responses. The Cochran-Mantel-Haenszel chi-squared test for these 2 categories revealed no statistically significant difference in the discontinuations at visit 2 between the 2 groups.

Forty-seven percent of the subjects were white and 47% were black. The mean age of the subjects was 30.6 years (range 18–49 years). Approximately 60% of the subjects had previously been treated for BV. The treatment compliance was determined by the use of at least 25 g of medication, which is 50% of the amount expected to be used. All evaluable patients who returned their medication tubes met the criteria for treatment compliance. Data are missing for 2 metronidazole and 2 triple-sulfa patients who did not return the study medication. The mean weight of medication was 56.3 g (range 33.5–70.4 g) for the metronidazole treatment group compared with 56.1 g (range 28.4–74 g) for the triple-sulfa treatment group.

Of the 237 evaluable patients, 205 were included in the final efficacy evaluation: 96 in the metronidazole group and 109 in the triple-sulfa group. The evaluable-for-efficacy set was composed of those patients who were evaluable at the final visit (visit 3) plus those who were evaluable at visit 2 and diagnosed as treatment failures. According to the protocol, efficacy was determined at 30 days after therapy (visit 3). The patients considered failures at visit 2 were dropped and included as failures for visit 3. Because of this design, an intent-to-treat analysis can be viewed in 2 ways: (method 1) inclusion of patients with data who had clinical evaluations at visit 3 but were considered nonevaluable at visit 3 because of a protocol exclusion plus patients with data at visit 2 who were treatment failures, but also were considered nonevaluable at visit 2 because of a protocol exclusion or (method 2) inclusion of all patients at visit 3. Both methods were analyzed, but only the results analyzed by method 1 are presented because the results were identical, regardless of the method used. Eight metronidazole-treated patients and 4 triple sulfa-treated patients were added to the efficacy set chosen. Of the 96 patients considered evaluable at visit 3 in the metronidazole treatment group, 22 had no third clinic visit because they were dropped at visit 2 as treatment failures. Therefore, the 74 patients in this group who were clinically evaluated at visit 3 had a mean of 37.9 days in the study (range 29–68 days). In the triple-sulfa treatment group, 32/109 patients considered evaluable at visit 3 had no third visit because they were dropped at visit 2 as treatment failures. One patient failed treatment at visit 2 but was not dropped. This patient, who returned for visit 3, was included in the group that returned for visit 3. Therefore, 77 patients in this group were clinically evaluated at visit 3, with a mean of 37.2 days in the study (range 24–57 days). One patient missed visit 2 but completed visit 3 ($P$ = NS).

At the first return visit, 81/103 (79%) subjects in the metronidazole treatment group were successfully treated compared with 80/113 (71%) in the triple-sulfa treatment group ($P = 0.33$). There were 63/96 (66%) subjects who were treatment successes at visit 3 for the metronidazole treatment group compared with 51/109 (47%) for the triple-sulfa treatment group ($P = 0.02$). When the individual criteria for BV (clue cells, pH, KOH amine odor test, homogeneous discharge) were evaluated, metronidazole was statistically superior to triple sulfa in eliminating 3 of the 4 criteria (clue cells, pH, KOH amine odor test) at the final visit ($P < 0.05$).

Gram's stains were obtained during 617/631 visits. A weighted score was determined based on the presence of bacterial morphotypes known to be positively or negatively associated with BV in which a score of 7–10 corresponded to BV. A score of 0–3 corresponded to normal or cured status and 4–6 reflected intermediate flora.[19] The mean Gram's stain score at study entry was 7.9 in the metronidazole group and 7.8 in the triple-sulfa group. The mean Gram's stain score at the final visit (either first or second return visit) in the metronidazole group was 3.4, for a mean reduction of 4.5, compared with the study entry score ($P < 0.001$). The mean Gram's stain score reduction in the triple-sulfa group from 7.8 at entry to 4.6 at the final visit was 3.2 ($P < 0.001$). The difference in mean Gram's stain scores of cured patients in the 2 treatment groups (4.5 for metronidazole and 3.2 for triple sulfa) was not statistically significant ($P > 0.5$). The clinical diagnoses in both groups at all their visits had high correlation (88%) with independent assessments by Gram's stains.

Forty-one patients in the metronidazole group and 30 in the triple-sulfa group reported mild adverse events related to therapy involving mainly the gastrointestinal and genitourinary tract ($P = 0.14$). Vaginal candidiasis occurred in 3 metronidazole and 2 triple-sulfa patients. One metronidazole patient reported a severe headache questionably attributable to topical metronidazole.

### DISCUSSION

For almost 20 years, oral metronidazole has been considered the treatment of choice for BV.[2–7,10] Although the therapeutic success rate with oral metronidazole at 1 month is 60–80%, the topical treatment of BV may be preferable to systemic therapy in some patients because of the frequency of unpleasant gastrointestinal side effects from oral metronidazole. In addition, hematologic and neurologic complications of oral therapy may accompany prolonged therapy. Ours is the first comparative therapeutic study of a double-blinded approach to BV with 2 topical drugs, 0.75% metronidazole gel and triple-sulfa cream. In the past, double-blinded studies compared oral with topical agents[9] or topical drug with placebo.[13,14]

In the current study, the cure rate of BV with intravaginal 0.75% metronidazole gel was superior to the rate with intravaginal triple-sulfa cream at the final visit (66% vs. 47%, $P = 0.02$). When the individual criteria for BV (clue cells, pH, KOH amine odor test, homogeneous discharge) were evaluated, metronidazole was statistically superior to triple-sulfa in eliminating 3 of the 4 criteria (clue cells, pH, KOH amine odor test) at the final visit ($P < 0.05$). The clinical diagnoses in the entire study including all investigators had high correlation (88%) with independent assessments by Gram's stains.

The overall success rate for topical metronidazole in this study was 5–10% lower than that observed in other trials using topical metronidazole gel.[8,16] These differences however, may be attributed to variability in the population or vaginitis severity that frequently exists among trials as well as to variability in the criteria for defining cure rather than to any true difference in the efficacy of metronidazole gel. In the current study, 60% of the patients had previously been treated for BV. In addition, 40% of the evaluable patients were enrolled by 2 investigators (J.D.S., J.T.) with clinical practices serving patients with chronic or intractable vaginitis.

The cure of BV at the third visit (≥29 days after the start of therapy) for women given intravaginal triple-sulfa was significantly less than the cure in patients given metronidazole gel. In fact, only 47% of the triple-sulfa group appeared to have clinical responses. However, this response rate is not as high as it seems when the response of BV to vaginal placebo is considered. Intravaginal placebo appeared to cure 40% of the cases of BV at 21 days after therapy in an earlier study.[19] In the same study, BV was cured at 21 days in 44% of the users of intravaginal triple-sulfa cream compared with 91% of tinidazole users ($P = 0.01$).[19] Tinidazole has the same spectrum of activity as metronidazole. In another study, only 14% of the patients with BV responded to triple-sulfa cream.[1]

The ineffectiveness of triple-sulfa vaginal cream in the treatment of BV is not difficult to understand. *Gardnerella vaginalis* requires folic acid for growth because it does not have the ability to change para-aminobenzoic acid (PABA) to folic acid. On the other hand, sulfa drugs act by the competitive inhibition of PABA to folic acid and only inhibit bacteria that utilize the PABA-folic-acid pathway. In fact, *G. vaginalis* is very resistant to sulfa.[20] The resistance is so marked that sulfa is used to distinguish *G. vaginalis* from other bacteria.[21] Anaerobic gram-negative rods are also not very sensitive to sulfa in vitro, which accounts for the high rate of anaerobe isolation following the use of triple-sulfa for BV.[1,19] This observation may explain the difference in recurrence rates after 1 month in the present study between the 2 groups, 11/74 (15%) and 25/75 (33%) for the metronidazole and triple-sulfa groups, respectively.

In preparing the manuscript, we reviewed the literature and found no reports of the treatment of BV with triple-sulfa cream since the standardization of the clinical diagnostic criteria by Amsel et al.[17] Triple-sulfa, commonly used to treat nonspecific vaginitis, continues to be extensively used. The present study indicates reasonably high immediate rates of symptomatic relief with triple-sulfa but considerably poorer long-term therapeutic efficacy.

The initial safety concerns associated with the use of systemic metronidazole in terms of carcinogenic effects in rodents[22] were found to be dose related. Although this possibility is of concern, there is no proof of carcinogenicity.[23,24] Nevertheless, serious side effects are well described.[6] Topical 0.75% metronidazole gel intravaginal therapy is associated with a limited absorption of metronidazole and reduced frequency of systemic adverse reactions, as observed in our study and other reports.[8,16] Local side effects and complications were uncommon in both treatment groups of our study.

In conclusion, 0.75% metronidazole vaginal gel was superior to triple-sulfa cream in the treatment of BV when the patients were evaluated at approximately 30 days after therapy. The adverse events with metronidazole were similar to those reported with triple-sulfa, and none of the metronidazole events were serious or severe. Triple-sulfa cream is not particularly efficacious in eradicating BV.

### ACKNOWLEDGMENTS

This work was supported by a grant from Curatek Pharmaceuticals, Limited Partnership, Elk Grove Village, IL.

### REFERENCES

1. Pheiffer TA, Forsyth PS, Durfee MA, et al.: Non-specific vaginitis: Role of *Haemophilus vaginalis* and treatment with metronidazole. N Engl J Med 298:1429–1434, 1978.
2. Hillier S, Holmes KK: Bacterial vaginosis. In Holmes KK, Mardh P, Sparling F (eds): Sexually Transmitted Diseases. 2nd ed. New York: McGraw-Hill, pp 547–559, 1989.
3. Bistoletti P, Fredricsson B, Hagstrom B, Nord CE: Comparison of oral and vaginal metronidazole therapy for nonspecific bacterial vaginosis. Gynecol Obstet Invest 21:144–149, 1986.
4. Eschenbach DA, Critchlow CW, Watkins H, et al.: A dose-duration study of metronidazole for the treatment of nonspecific vaginosis. Scand J Infect Dis Suppl 40:73–80, 1983.
5. Blackwell Al, Fox AR, Phillips I, Barlow D: Anaerobic vaginosis (nonspecific vaginitis): Clinical, microbiological and therapeutic findings. Lancet 2:1379–1382, 1983.
6. Lugo-Miro VI, Green M, Mazur L: Comparison of different metronidazole therapeutic regimens for bacterial vaginosis. A meta-analysis. JAMA 268:92–95, 1992.
7. Boeke AJ, Dekker JH, van Eijk JT, et al.: Effect of lactic acid suppositories compared with oral metronidazole and placebo in bacterial vaginosis: A randomized clinical trial. Genitourin Med 69:388–392, 1993.
8. Livengood CH, McGregor JA, Soper DE, et al.: Bacterial vaginosis: Efficacy and safety of intravaginal metronidazole treatment. Am J Obstet Gynecol 170:159–164, 1994.
9. Schmitt C, Sobel JD, Meriwether C: Bacterial vaginosis: Treatment with clindamycin cream versus oral metronidazole. Obstet Gynecol 79:1020–1023, 1992.
10. McDonald HM, O'Loughlin JA, Vigneswaran R, et al.: Bacterial vaginosis in pregnancy and efficacy of short-course oral metronidazole treatment: A randomized controlled trial. Obstet Gynecol 84:343–348, 1994.
11. Greaves WL, Chungafung J, Morris B, et al.: Clindamycin versus metronidazole in the treatment of bacterial vaginosis. Obstet Gynecol 72:799–802, 1988.
12. Livengood CH III, Thomason JL, Hill GB: Bacterial vaginosis: Diagnostic and pathogenic findings during topical clindamycin therapy. Am J Obstet Gynecol 163: 515–520, 1990.
13. Livengood CH III, Thomason JL, Hill GB: Bacterial vaginosis: Treatment with topical intravaginal clindamycin phosphate. Obstet Gynecol 76:118–123, 1990.
14. Hillier SL, Krohn MA, Watts DH, Wölner-Hanssen P, Eschenbach DA: Microbiologic efficacy of intravaginal clindamycin cream for the treatment of bacterial vaginosis. Obstet Gynecol 76:407–413, 1990.
15. Biswas MK: Bacterial vaginosis. Clin Obstet Gynecol 36:166–176, 1993.
16. Hillier SL, Lepenski CM, Briselden AM, Eschenbach DA: Efficacy of intravaginal 0.75% metronidazole gel for the treatment of bacterial vaginosis. Obstet Gynecol 81: 963–967, 1993.
17. Amsel R, Totten PA, Spiegel CA, Chenk CS, Eschenbach D, Holmes KK: Nonspecific vaginitis. Am J Med 74:14–27, 1983.
18. Nugent RP, Krohn MA, Hillier SL: Reliability of diagnosing bacterial vaginosis is improved by a standardized method of Gram stain interpretation. J Clin Microbiol 29:297–301, 1991.
19. Piot P, Van Dyck E, Godts P, Vanderheyden J: A placebo-controlled, double-blind comparison of tinidazole and triple sulfonamide cream for treatment of non-specific vaginitis. Am J Obstet Gynecol 147:85–89, 1983.
20. McCarthy LR, Mickelsen PA, Smith EG: Antibiotic susceptibility of *Haemophilus vaginalis* (*Corynebacterium vaginale*) to 21 antibiotics. Antimicrob Agents Chemother 16:186–190, 1979.
21. Bailey RK, Voss JL, Mith RF: Factors affecting isolation and identification of *Haemophilus vaginalis* (*Corynebacterium vaginale*). J Clin Microbiol 9:65–68, 1979.
22. Rustia M, Shubik P: Induction of lung tumors and malignant lymphomas in mice by metronidazole. J Natl Cancer Inst 48:721–729, 1972.
23. Roe FJC: Toxicologic evaluation of metronidazole with particular reference to carcinogenic, mutagenic, and teratogenic potential. Surgery 93:158–164, 1983.
24. Beard CM, Noller KL, O'Fallon WM, et al.: Lack of evidence for cancer due to use of metronidazole. N Engl J Med 301:519–522, 1979.
